# Studying bacteria in respiratory specimens by using conventional and molecular microbiological approaches

**DOI:** 10.1186/1471-2466-9-14

**Published:** 2009-04-15

**Authors:** Geraint B Rogers, Thomas WV Daniels, Andrew Tuck, Mary P Carroll, Gary J Connett, Gondi JP David, Kenneth D Bruce

**Affiliations:** 1 King's College London, Molecular Microbiology Research Laboratory, Pharmaceutical Science Division, 150 Stamford Street, Franklin-Wilkins Building, King's College London, London, SE1 9NH, UK; 2 Lung Transplant Unit, Dept of Thoracic Medicine, Prince Charles Hospital, Rode Road, Chermside, Brisbane, Queensland 4032, Australia; 3 Health Protection Agency South East, Southampton Laboratory, Level B, South Laboratory Block, Southampton General Hospital, Southampton, S016 6YD, UK; 4 Cystic Fibrosis Unit, Southampton University Hospitals NHS Trust, Tremona Road, Southampton, SO16 6YD, UK; 5 Department of Paediatrics, Southampton University Hospitals NHS Trust, Southampton S016 6YD, UK

## Abstract

**Background:**

Drawing from previous studies, the traditional routine diagnostic microbiology evaluation of samples from chronic respiratory conditions may provide an incomplete picture of the bacteria present in airways disease. Here, the aim was to determine the extent to which routine diagnostic microbiology gave a different assessment of the species present in sputa when analysed by using culture-independent assessment.

**Methods:**

Six different media used in routine diagnostic microbiology were inoculated with sputum from twelve patients. Bacterial growth on these plates was harvested and both RNA and DNA extracted. DNA and RNA were also extracted directly from the same sample of sputum. All nucleic acids served as templates for PCR and reverse transcriptase-PCR amplification of "broad range" bacterial 16S rRNA gene regions. The regions amplified were separated by Terminal Restriction Fragment Length Polymorphism (T-RFLP) profiling and compared to assess the degree of overlap between approaches.

**Results:**

A mean of 16.3 (SD 10.0) separate T-RF band lengths in the profiles from each sputum sample by Direct Molecular Analysis, with a mean of 8.8 (SD 5.8) resolved by DNA profiling and 13.3 (SD 8.0) resolved by RNA profiling. In comparison, 8.8 (SD 4.4) T-RF bands were resolved in profiles generated by Culture-derived Molecular Analysis. There were a total of 184 instances of T-RF bands detected in the direct sputum profiles but not in the corresponding culture-derived profiles, representing 83 different T-RF band lengths. Amongst these were fifteen instances where the T-RF band represented more than 10% of the total band volume (with a mean value of 23.6%). Eight different T-RF band lengths were resolved as the dominant band in profiles generated directly from sputum. Of these, only three were detected in profiles generated from the corresponding set of cultures.

**Conclusion:**

Due to their focus on isolation of a small group of recognised pathogens, the use of culture-dependent methods to analyse samples from chronic respiratory infections can provide a restricted understanding of the bacterial species present. The use of a culture-independent molecular approach here identifies that there are many bacterial species in samples from CF and COPD patients that may be clinically relevant.

## Background

Sputum culture has been used by the respiratory physician to provide insight into the bacteria present in many airway diseases such as pneumonia, Cystic Fibrosis (CF) and Chronic Obstructive Pulmonary Disease (COPD) [1,2]. In COPD for example, the presence of bacteria in the lower airways has been correlated with exacerbation frequency [3], airways inflammation [4], and indirectly with decline in lung function [5], Moreover in CF, the first identification of *Pseudomonas aeruginosa *from the lower airways has been negatively correlated with decline in lung function and survival [6,7]. Despite this, the results of conventional sputum culture and sensitivity tests are often not used to alter management in the chronic phase of these conditions [8]. To exemplify this, in a review of outcomes following pulmonary exacerbations in the placebo control arm of a large inhaled tobramycin trial, Smith *et al *[9] found CF patients with resistant *P. aeruginosa *fared no worse than those with sensitive strains (both groups were treated with standardised intravenous antibiotics). Furthermore, for such a key diagnostic tool, it would be hoped that the conventional sputum culture and sensitivity tests as performed by routine Diagnostic Microbiology Laboratories would have good measures of inter- and intra-operator reproducibility. For CF sputum analysis at least, Foweraker *et al *have demonstrated this not to be the case [10].

Thus, despite the central importance of bacteria to pulmonary medicine, this standard tool for bacterial identification does not appear to be useful or to perform as well as would be desired. To environmental microbiologists, this may not come as a surprise. In environments such as soil and sea water, most of the bacteria present cannot be cultured [11]. The process of the derivation of pure cultures *in vitro *on solidified medium prior to identification, as first developed by Robert Koch in the late nineteenth century [12], is still however the means by which routine Diagnostic Microbiology analyses clinical samples. Conceptually, there appears to be no reason why bacteria that inhabit the environment of the human lung should be necessarily different from this culture bias. Routine diagnostic microbiology uses specific growth protocols to isolate species considered to be significant in disease. Whilst this process can provide efficient assays for known aetiological agents, when applied uncharacterised, mixed infections, it can preclude the identification of novel pathogens and species that would not typically be expected in airway samples. As such, it is important to develop at very least parallel systems of analysis.

One such approach has used nucleic acids extracted directly from clinical samples to detect and identify bacterial species. In this culture-independent approach, nucleic acid extracts serve as templates for the PCR amplification of 16S ribosomal RNA genes spanning all Bacteria ("broad range") [13]. This PCR uses conserved regions of the gene to serve as primers, with the variable sequence between these primer sites serving to identify the bacterial species. One such method, Terminal Restriction Fragment Length Polymorphism (T-RFLP) analysis, resolves multiple bacterial species in a single sample as a discrete set of bands formed by the species-characteristic lengths of the first cut position of a single restriction endonuclease in ribosomal gene PCR products [14]. Through comparison to predicted cut lengths, the bacterial species in a sample can be assigned tentatively as a series of species identities. We have previously used this approach to study the bacterial communities present in CF sputum [15,16], with the most abundant (or dominant) species present identified through the analysis of the intensity and width of each band formed. Also, the presence of metabolically active bacteria can be detected through the reverse transcription of 16S rRNA extracted and analysed also by T-RFLP (RT-T-RFLP). Again, previously we have used this approach to show that CF sputum samples contain metabolically active bacteria [17].

In this study, we extend this work to compare the bacterial species detected in twelve CF and COPD sputum samples by culture-dependent and culture independent analysis. Each sample was divided in two. One portion was analysed directly by T-RFLP and RT-T-RFLP (Direct Molecular Analysis). The other portion was cultured *in vitro *on a selection of media that would form the typical range used for respiratory samples by a diagnostic microbiology laboratory. To allow comparison of species recovered, all culture plate growth was also analysed by both T-RFLP and RT-T-RFLP (Culture-derived Molecular Analysis). The substantial differences found in terms of species detected by culture-dependent and independent strategies are discussed subsequently.

## Methods

### Sample collection and processing

Sputum samples were obtained from 8 adult CF and 4 COPD patients attending Southampton General Hospital, Hampshire, UK and Lymington Hospital (Table 1). All patients were clinically stable at the time of sampling and had not received antibiotic treatment for 30 days. Samples divided into two aliquots, one of which was stored immediately at -80°C and other of which was used to inoculate a range of growth media. Cultures were performed in accordance with standard Health Protection Agency laboratory practices [18]. The growth media used were blood agar (referred to here as BLOOD), Cysteine Lactose-Electrolyte-Deficient (CLED), a selective medium for Gram-positive bacteria (CNA), chocolate agar (CHOC), selective medium for *Pseudomonas *species (PYO), selective medium for fungi and yeasts (SNA) and a selective medium for *B. cepacia *complex (CEP). All media were supplied by E & O Laboratories Limited, Burnhouse, Bonnybridge, Scotland.

**Table 1:** Background clinical in formation regarding the patients involved in the study.

**patient**	**condition**	**FEV_**1 **_(% predicted)**	**age**
1	CF	60%	30
2	CF	57%	45
3	CF	37%	47
4	CF	35%	22
5	CF	49%	55
6	CF	45%	21
7	CF	48%	40
8	CF	20%	22
9	COPD	60%	77
10	COPD	14%	69
11	COPD	16%	74
12	COPD	37%	70

FEV_1 _represents the volume of air that can be forced out in 1 second after taking a deep breath, a commonly used measure of lung function.

All cultures were handled and incubated in accordance with routine microbial surveillance practices. Following incubation, all growth present on the culture media was scraped off and placed in sterile tubes with 2.5 ml 0.9% saline (a separate tube being used for each culture plate). These tubes were stored at -80°C prior to nucleic acid extraction.

### Nucleic acid extraction

Prior to DNA extraction, sputum samples were washed in sodium phosphate buffer to remove adherent saliva. DNA and RNA extraction from sputum samples and the cultured organisms was then carried out as previously described [16].

All reagents, glassware and plastics used in RNA work were DEPC-treated prior to use. RNA was extracted as follows: 0.75 ml of Tri Reagent (Sigma-Aldrich, Dorset, UK) were added to approximately 0.2 ml of each sample and vortexed for 1 min. Samples were incubated at room temperature for 5 min prior to the addition of 0.2 ml chloroform. Samples were vortexed for 15 sec. and incubated at room temperature for 5 min. Phases were separated by centrifugation at 12,000 × *g *for 15 min at 4°C.

### Isolation of DNA

0.3 ml of 100% ethanol was added to precipitate the DNA from the lower phase. The sample was mixed by inversion, incubated at room temperature for 3 min and centrifuged at 12,000 × *g *for 5 min at 4°C. The pellet was washed in 0.1 M sodium citrate, 10% ethanol solution (during each wash the pellet was allowed to stand for at least 30 min). Pellets were centrifuged at 12,000 × *g *for 5 min at 4°C and washed twice in 75% ethanol. The DNA was vacuum dried, with the pellet resuspended in 50 μl H_2_O and stored at -20°C.

### Isolation of RNA

The upper phase was transferred to a fresh microfuge tube and 0.5 ml of propan-2-ol was added. Samples were incubated for 10 min at room temperature and RNA was pelleted by centrifugation at 12,000 × *g *for 10 min at 4°C. The supernatant was removed and the RNA pellet washed once in 75% ethanol and re-pelleted by centrifugation at 7,500 × g for 5 min at 4°C. Pellets were air-dried for 10 min, resuspended in 30 μl distilled water and incubated for 10 mins at 55°C. Purified RNA samples were stored as aliquots at -70°C. Prior to reverse transcription, any residual DNA was removed using DNAseI (Epicentre, Madison, USA) in accordance with the manufacturer's instructions, with PCR amplification controls performed as appropriate.

### Reverse transcription

cDNA was generated from the isolated RNA using the reverse primer 926r (see below) and AMV reverse transcriptase (Promega, Southampton, UK) in accordance with the manufacturer's instructions. Double stranded DNA was generated using 1 μl of this cDNA as template in a 50 μl PCR reaction containing both primers (8f700 and 926r). PCR products amplified were verified by Tris-Acetate- EDTA (TAE)-agarose gel electrophoresis on 0.8% (wt/vol) TAE-agarose gels stained in ethidium bromide (0.5 mg/L) with images, viewed on a UV transilluminator (Herolab, Wiesloch, Germany), captured by using a Herolab image analyser with E.A.S.Y STOP win 32 software (Herolab).

### PCR amplification and restriction endonuclease digestion

The oligonucleotide primers used to amplify a region of the 16S rRNA gene for members of the Domain Bacteria, 8f700 (5'-AGA GTT TGA TCC TGG CTC AG-3') and 926r (5'-CCG TCA ATT CCT TTR AGT TT-3') were as described previously [14]. Primer 8f700 was labelled at the 5' end with IRD700 (TAGN, Gateshead, UK); primer 926r was unlabeled. PCR mixtures comprised 1× PCR buffer, 1.5 mM MgCl_2_, each deoxynucleoside triphosphate at a concentration of 0.2 mM, each primer at a concentration of 0.2 mM, and 1 U of REDTaq DNA polymerase (Sigma-Aldrich, Gillingham, UK), in a final volume of 50 μl. The final concentration of the template DNA used was approximately 50 ng. An initial denaturation step of 94°C for 2 min was followed by 32 cycles of denaturation at 94°C for 1 min, annealing at 56°C for 1 min, and extension at 72°C for 2 min, with a final extension step at 72°C for 10 min. Amplification was carried out by using a GeneAmp PCR System 2400 (Perkin-Elmer, Beaconsfield, UK). PCR products amplified were verified as described above.

PCR products (ca. 200 ng) were digested by using the restriction endonuclease *Cfo*I (Roche, Lewes, United Kingdom) for 3 h at 37°C with the reaction buffer supplied by the manufacturer. All restriction endonuclease digestions were carried out to complete digestion as shown by comparing PCR products after various digestion incubation times (data not shown). The restriction endonuclease was inactivated by heating at 90°C for 20 min. An approximately 100 ng portion of digested PCR products for T-RFLP analysis was separated by length by using a 25 cm SequagelXR denaturing polyacrylamide gel (National Diagnostics) prepared in accordance with the manufacturer's instructions, with the addition of 8.3 M urea and 10% (final concentration v/v) formamide, using a LI-COR IR2 automated DNA sequencer (LI-COR Biosciences) at 55°C and 1,200 V.

### T-RFLP profile analysis

T-RFLP profiles were analyzed using Phoretix 1D Advanced software v.5.10 (Nonlinear Dynamics, Newcastle upon Tyne, UK). The sizes of the bands resolved by T-RFLP were determined by comparing their relative position with known size markers, comprised of bands equivalent to 75, 100, 150, 200, 250, 300, 350, 400, 450, 500, 600, 700, 800, 900 and 1000 bases of single-stranded DNA (microSTEP 15a [700 nm], Microzone, Lewes, UK). Phoretix 1D Advanced software was also used to determine the volume of each band (with band volume the product of the area over which a band was detected and the intensity of signal recorded over that area). Band volume was expressed as a percentage of the total volume of bands detected in a given electrophoretic profile. T-RFLP bands were resolved over the region between 50 and 958 bases. No bands shorter than 50 bases in length were recorded as they were in the region susceptible to high levels of signal stemming from the IR tag on unattached 8f700IR primer. In this study, the threshold used to detect bands was 0.01% of the total signal between the 50 and 958 base region.

## Results

### Direct Molecular Analysis – overall assessment

**Figure 1:**
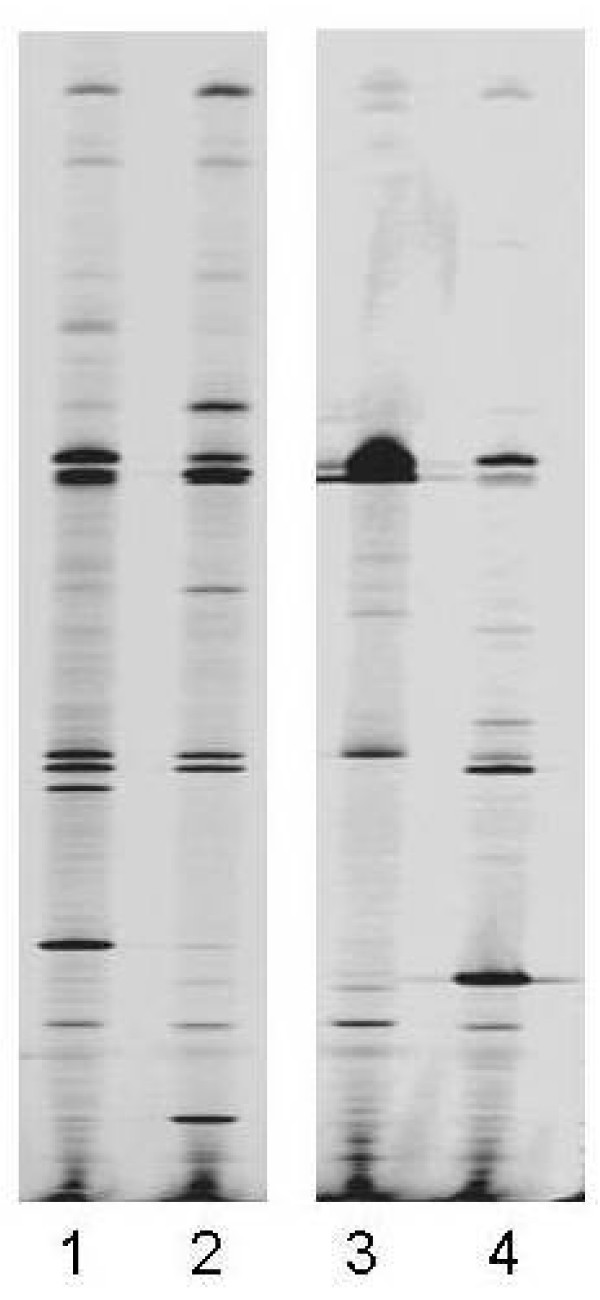
**Two examples of pairs of DNA profiles generated from two sputum samples (A and B), one from Direct Molecular Analysis and one from the total pool of colonies isolated from that sputum using routine surveillance media**. Lane 1 – sample A, Culture-derived Molecular Analysis, Lane 2 – sample A, Direct Molecular Analysis, Lane 3 – sample B, Culture-derived Molecular Analysis, Lane 4 – sample B, Direct Molecular Analysis.

**Figure 2:**
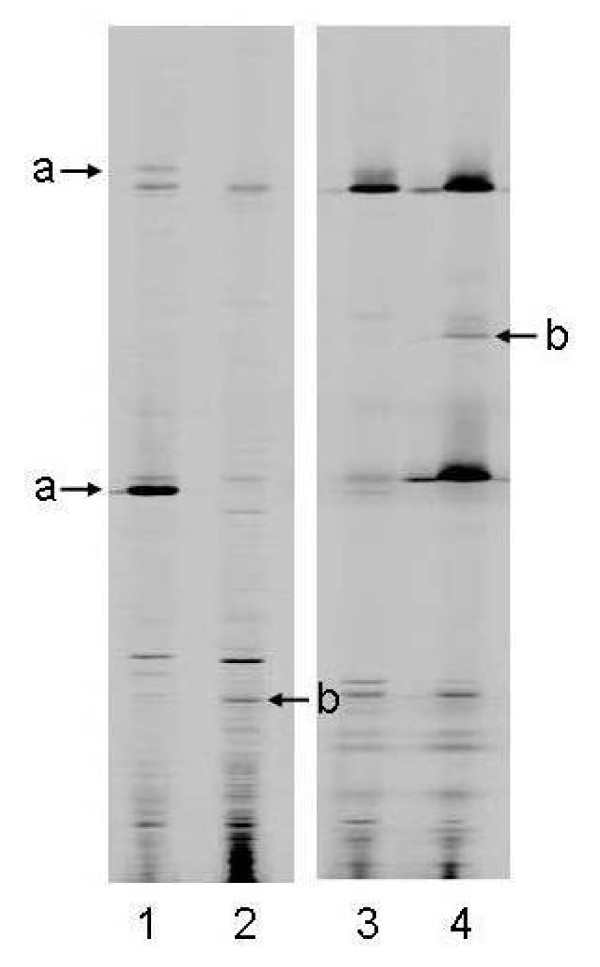
**Two examples of pairs of DNA and RNA profiles generated from two sputum samples (C and D), by Direct Molecular Analysis**. Lane 1 – sample C, DNA, Lane 2 – sample C, RNA, Lane 3 – sample D, DNA, Lane 4 – sample D, RNA. Arrows marked "a" indicate two examples where bands are present in the DNA profile but absent from the RNA profile. Arrows marked "b" indicate two examples where bands are present in the RNA profile but absent from the DNA profile.

Examples of the ways in which T-RFLP profiles, generated either directly from sputum samples (Direct Molecular Analysis), or from cultures derived from those samples (Culture-derived Molecular Analysis), differ are shown in Figure 1. Here, each T-RFLP profile contains a varying number of T-RF bands of different lengths. T-RF bands also allow an assessment of the relative abundance of the species present. Examples of the ways in which profile patterns differ depending on whether they are generated from the DNA or RNA component of a sample are shown in Figure 2. These differences in T-RF band intensities can be used to obtain a relative measure of metabolic activity.

**Figure 3:**
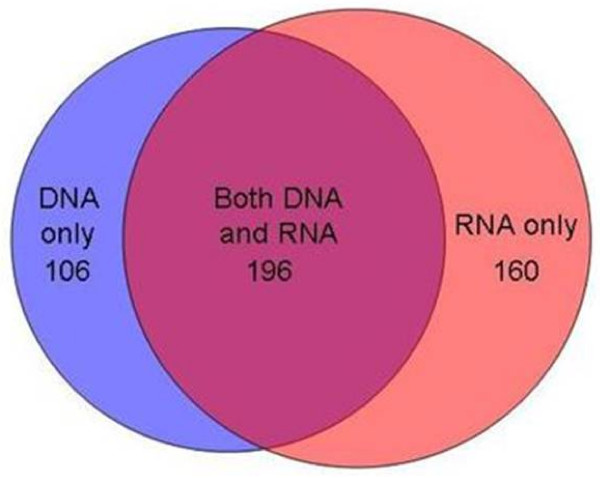
**Venn diagram showing the overlap between the T-RF bands detected in the DNA and RNA profiles generated by Direct Molecular Analysis from sputum samples**.

A breakdown of numbers of T-RF bands resolved in the DNA-derived and RNA-derived profiles generated from the sputum samples directly and following cultivation on different media are shown in Table 2. Overall, a total of 266 T-RF bands were resolved in the DNA and RNA-derived T-RFLP Direct Molecular Analysis profiles generated directly from these sputum samples, representing 104 different T-RF band lengths. The most frequent band length was identified 19 times, with 66 band lengths found only once. Of these 266 T-RF bands, a total of 106 were resolved in the DNA-based T-RFLP profiles alone, representing 65 different T-RF band lengths. 196 T-RF bands were detected in both the DNA and RNA-derived profiles, representing 96 different band lengths, and a total of 160 bands were detected in the RNA-based T-RFLP profiles alone representing 79 band lengths as shown schematically in Figure 3.

**Table 2:** Number of T-RF bands resolved in T-RFLP profiles generated from the culture-independent and culture-based approaches.

		**Direct Molecular Analysis**			**Culture-derived Molecular Analysis**														
**Patient**	**Condition**	**DNA**	**RNA**	**Total T-RFs**	**CLED**		**PYO**		**CHOC**		**BLOOD**		**CEP**		**SNA**		**CNA**		**Total T-RFs**
						
					**DNA**	**RNA**	**DNA**	**RNA**	**DNA**	**RNA**	**DNA**	**RNA**	**DNA**	**RNA**	**DNA**	**RNA**	**DNA**	**RNA**	

1	CF	23	23	32	3	3	8	7	8	7	3	3	0	0	0	0	3	3	19
2	CF	3	10	10	3	2	2	2	NA	NA	3	2	2	2	0	0	3	3	6
3	CF	3	5	6	2	2	2	2	2	2	2	2	2	2	0	0	2	2	2
4	CF	3	3	3	3	3	3	1	3	5	2	3	2	2	0	0	5	8	11
5	CF	15	18	21	2	2	2	2	4	4	3	6	0	0	0	0	3	2	8
6	CF	7	8	9	2	2	3	3	3	4	4	4	2	2	0	0	2	4	4
7	CF	6	7	7	3	3	2	2	2	2	5	4	0	0	0	0	2	2	6
8	CF	10	19	27	4	4	6	6	4	4	0	0	2	2	2	2	3	5	12
9	COPD	6	9	13	NA	NA	NA	NA	2	2	2	5	NA	NA	NA	NA	NA	NA	8
10	COPD	11	10	17	NA	NA	NA	NA	2	2	5	7	NA	NA	NA	NA	NA	NA	11
11	COPD	8	19	19	NA	NA	NA	NA	9	8	3	3	NA	NA	NA	NA	NA	NA	11
12	COPD	11	29	32	NA	NA	NA	NA	5	5	4	4	NA	NA	NA	NA	NA	NA	8

CLED – Cysteine Lactose-Electrolyte-Deficient agar, PYO – selective medium for *Pseudomonas *species, CHOC – chocolate agar, BLOOD – blood agar, CEP – selective medium for *B. cepacia *complex, SNA – selective medium for fungi and yeasts, CNA – selective medium for Gram-positive bacteria.

### Direct Molecular Analysis – assessment per sample

On average, 8.8 (Standard Deviation 5.8) and 13.3 (SD 8.0) T-RF bands were resolved from the DNA and RNA isolated directly from sputum respectively, representing a mean of 16.3 (SD 10.0) separate T-RF band lengths in the profiles from each sputum sample. On average, 5.7 (SD 4.1) T-RF bands were detected in both the DNA and the RNA profile from the sample set as a whole, 3.2 (SD 3.4) were detected in the DNA profile alone, and 7.6 (SD 6.3) were detected in the RNA profile alone.

### Culture-derived Molecular Analysis

A composite of "all" Culture-derived Molecular Analysis T-RF bands was formed representing the species detected on all types of media. Within this, a total of 40 different T-RF band lengths were resolved. In the sample set, an average of 2.9 separate T-RF lengths were detected in the profiles generated from the CLED agar, 3.5 from PYO agar, 4.3 from chocolate agar, 4.7 from BLOOD agar, 1.3 from CEP agar, 0.4 from SNA agar, 3.8 from CNA agar. The highest number of separate band lengths was resolved from the bacteria cultured on chocolate agar (21), followed by BLOOD agar (18), CNA agar (15), PYO agar (13), CLED agar (10), CEP agar (4) and SNA agar (1). On average, 8.8 (SD 4.4) separate T-RF bands were resolved by Culture-derived Molecular Analysis per patient.

### Routine diagnostic microbiology

Analysis of "historical" culture-based routine diagnostic microbiology surveillance data revealed that, for these 12 patients, eight were reported as being infected with *Pseudomonas *spp., with six reported as having (normal) oral flora. *Escherichia coli *and *Staphylococcus aureus *were each reported in a single case.

### Comparing the approaches

The difference in the average number of T-RF bands detected in the samples between the Direct Molecular Analysis and Culture-derived Molecular Analysis groups was determined to be highly statistically significant (P = 0.008, two-tailed paired T-test). For 77.9% (SD 20.9%) of the T-RF band lengths resolved from the Culture-derived Molecular Analysis, a band of the same length was detected at the same position in both the DNA-derived and RNA-derived T-RFLP profiles from the same sample. In 15.4% (SD 16.8%) of instances, a band was detected in the RNA profile alone, and in 6.7% (SD 7.2%) of instances, a band was detected in the DNA profile alone. Overall, of all the T-RF band lengths resolved from the Culture-derived Molecular Analysis, a match to at least one T-RF from DNA-derived or RNA-derived Direct Molecular Analysis was 92.5%. For comparison of the results obtained using the different strategies, see Table 3.

**Table 3:** Summary of a comparison of the results of Direct Molecular Analysis (DMA), Culture-derived Molecular Analysis (CMA), and Routine Diagnostic Microbiology (RDM)

**Patient**	**Condition**	Were all species reported in RDM represented in DMA?		Was the dominant band detected by DMA also detected by RDM?		Were there bands detected by DMA that were not detected by CMA?		Was the dominant band detected by DMA also detected CMA?		Was the dominant band detected by RT DMA also detected by CMA?	
		
		DNA	RNA	DNA	RNA	DNA	RNA	DNA	RNA	DNA	RNA
1	CF	N*	N*	N	N	Y	Y	Y	Y	Y	Y
2	CF	Y	Y	Y	Y	Y	Y	Y	Y	Y	Y
3	CF	Y	Y	Y	Y	Y	Y	Y	Y	Y	Y
4	CF	Y	Y	Y	Y	N	N	Y	Y	Y	Y
5	CF	Y	Y	N	Y	Y	Y	Y	Y	Y	Y
6	CF	Y	Y	Y	Y	Y	Y	Y	Y	Y	Y
7	CF	Y	Y	Y	Y	Y	Y	Y	Y	Y	Y
8	CF	Y	Y	Y	N	Y	Y	Y	Y	Y	Y
9	COPD	Y	Y	N	N	Y	Y	Y	Y	Y	Y
10	COPD	Y	Y	N/A	N/A	Y	Y	Y	Y	Y	Y
11	COPD	Y	Y	N/A	N/A	Y	Y	N	N	Y	Y
12	COPD	Y	Y	N/A	N/A	Y	Y	Y	Y	Y	Y

Totals (%)		11/12 (91.7)	11/12 (91.7)	6/9 (66.7)	6/9 (66.7)	11/12 (91.7)	11/12 (91.7)	11/12 (91.7)	11/12 (91.7)	12/12 (100)	12/12 (100)

* – species not represented in DMA – see text

**Figure 4:**
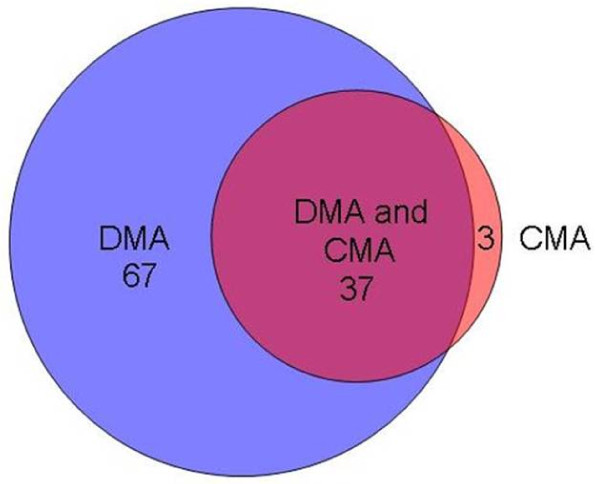
**Venn diagram showing the overlap between the T-RF bands detected in the profiles generated by Direct Molecular Analysis and those detected using Culture-derived Molecular Analysis**.

There were a total of 184 instances of T-RF bands detected in the direct sputum profiles alone, representing 83 different T-RF band lengths. Amongst these were fifteen instances where the T-RF band represented more than 10% of the total band volume for the profile (with a mean value of 23.6%). These instances were spread between 12 patients and represented 12 different T-RF band lengths. There were 46 instances of T-RF bands being resolved in the RNA profiles generated from cultures but having no corresponding band in the RNA-derived profile generated directly from the sputum sample. These T-RF bands represented 34 different band lengths. The degree to which the different T-RF band lengths were detected in the Direct Molecular Analysis and Culture-derived Molecular Analysis is illustrated in Figure 4.

The number of T-RF bands resolved in T-RFLP profiles generated from the culture-independent and culture-based approaches are shown in Table 2. In four instances, material cultured from inoculated bacterial media plates yielded no T-RF bands in either the DNA or the RNA profiles (Patient 8 – BLOOD agar, Patient 1 – CEP agar, Patient 5 – CEP agar, Patient 7 – CEP agar,). No T-RF bands were detected in either the DNA-derived or RNA-derived profiles from any of the SNA (yeast) agar, except in the case of Patient 8. In both the DNA-derived and RNA-derived profiles generated from Patient 8, two T-RF bands, both consistent with Pseudomonas species were detected. In one instance, a chocolate medium culture was not available for analysis (Patient 2).

Five different T-RF band lengths (155, 564, 582, 598, and 373 bases) were resolved as the most abundant ("dominant") band in DNA-derived profiles generated directly from sputum. The first four of these bands are consistent with those generated from *P. aeruginosa*, *Pseudomonas *sp., *Streptococcus constellatus *and *Lactobacillus *sp., respectively, as determined by analysis of published sequence data. It was not possible to assign an identity to the 373 base T-RF band due to multiple species being predicted to generate a band of this length. Strategies for resolving this problem are discussed below. Five different T-RF bands (155, 376, 564, 583, and 592 bases) were resolved in the equivalent RNA-derived profiles. These T-RF bands are consistent with those generated from *P. aeruginosa*, *Actinomyces *sp., *Pseudomonas *sp., *Streptococcus constellatus *and *Carnobacterium *sp., respectively. Overall, eight different T-RF band lengths were resolved as the most abundant ("dominant") band in profiles generated directly from sputum. Of these, only three were detected in profiles generated from the corresponding set of cultures (155, 373 and 376 bases).

On average, the most abundant T-RF band length detected in the DNA-derived profiles not resolved in any of the corresponding Culture-derived Molecular Analysis represented 34.0% (SD 29.3%) of the total signal volume. On average, the most abundant T-RF band length detected in the RNA-derived profiles not resolved in any of the corresponding Culture-derived Molecular Analysis represented 21.0% (SD 15.5%) of the total signal volume. The average volumes of bands detected in the DNA-derived and RNA-derived profiles, but not in the corresponding Culture-derived Molecular Analysis profiles were 9.0% (SD 17.0%) and 4.9% (SD 7.7%) respectively.

## Discussion

The process of bacterial culture *in vitro *has been shown to be selective and provides a distorted representation of the bacteria present in a clinical sample. Diagnostic microbiology has in fact for many years exploited this through the use of media for the selective growth of particular species or groups of species whilst excluding others leading to their isolation from complex contexts. This ability to exclude species makes diagnostic microbiology a useful tool when assaying for particular aetiological agents, such as those that are known to be responsible for some acute airway infections. However, when applied to the analysis of chronic airway infections, such as those associated with CF and COPD, such approaches may fail to identify the many opportunistic pathogens that could potentially colonise the airways. In an effort however to avoid biases associated with culture-based analysis, culture-independent techniques are being increasingly used to characterise bacteria found in chronic airways diseases. We set out to determine the extent to which routine diagnostic microbiological culture was masking the bacterial species present in a set of respiratory samples. Here, we report significant differences in the composition of bacterial communities in sputa as characterised by Direct Molecular Analysis and Culture-derived Molecular Analysis performed on the same samples. Differences both in the number and identity of the organisms resolved, their relative prevalence and their relative levels of metabolic activity were identified. Overall, these findings were considered as a series of questions that a respiratory physician would ask (Table 3) and are discussed in that fashion as below.

In terms of the bacteria "missed" by culture, the findings were marked. Overall, there were a total of 184 instances of bands detected in the Direct Molecular Analysis profiles but not in Culture-derived Molecular Analysis profiles, representing 83 different T-RF band lengths. Of course, this in itself does indicate whether these organisms were clinically significant, however, two factors suggest that they might be. Firstly, two of the five T-RF band lengths identified as dominant in T-RFLP profiles generated from sputum were not resolved in the profiles generated from the corresponding cultures. This suggests that in some cases, species that represent a significant proportion of the bacteria in the sample might be missed. Secondly, metabolically active bacteria, as detected by RT-T-RFLP profiling, were amongst those responsible for these dominant T-RF bands. Assuming that these were not acquired in transit through the upper airway [19], this means that metabolically active bacteria were present in the lower airways in significant numbers. As such, they are likely to have elicited an immune response of some form and may have potential roles as lung pathogens. Furthermore, such species are likely to be involved in complex inter-species communication that impacts on the bacterial community [20,21].

In relation to these marked differences, it is likely that one group that may be highly represented are those bacteria that require either anaerobic, microaerophilic or similar conditions for growth. No diagnostic microbiology service that we are aware of would routinely test for the presence of bacteria requiring anaerobic conditions for growth in respiratory samples of this kind. Despite this, the importance of micro-anaerobic environments within the lower airways of patients suffering from CF is being increasingly recognised [22,23]. This is all the more important given the wide range of clinically important anaerobic species, for example within genera such as *Bacteroides*, *Fusobacterium*, *Porphyromonas*, *Prevotella*, and *Peptostreptococcus*, that have previously been identified by 16S rRNA clone sequence analysis in respiratory samples from CF patients [16,24].

Whilst the assignment of species identities to T-RF band lengths was not the focus of this study, comparison of the T-RF band lengths generated from the different sample types allowed assessment of the degree to which data from culture-dependent and culture-independent methodologies overlapped. In only three cases, culture-based diagnostics generated a T-RF length that was not detected by molecular means. This is analogous to the situation regarding the results of the routine diagnostic microbiology where there was only one instance of a species being reported by conventional diagnostics that was not resolved by direct T-RFLP profiling. On this one occasion, T-RFLP profiling of Culture-derived Molecular Analysis resulted in the detection of a band tentatively identified as being *S. aureus*. There are many explanations of this including whether cultivation over-represents this species, the impact of detection thresholds in T-RFLP profiling, and possible contamination of growth media. Incorporating specific PCR based assays e.g. Alarcón *et al *[25] into the next phase of work will be valuable in determining the likely origin of such discrepancies. Equally however, to form such a small part of all the species present, this again raises important questions over the clinical significance of the other species that were much more common in the sample. Therefore, it must be questioned whether or not species that are "missed" by culture are really present at levels that make them of clinical significance. In relation to this, of the band lengths detected as the dominant band in either the DNA or RNA profiles generated directly from the sputum, only three of the 8 were detected in any of the corresponding Culture-derived Molecular Analysis profiles. The concept of over representation was taken further. It was found that the typical band volume of bands detected in the direct DNA profile, but not in culture, was approximately 10% of the total lane volume. This means that some of the most numerically significant species present in the sample are, in many cases, going undetected by culture.

The restriction enzyme, *Cfo*I, was selected because it is able to differentiate between the recognised key species associated with CF and COPD respiratory infections (*P. aeruginosa, S. aureus, B. cepacia, H. influenzae, S. maltophilia, S. pneumoniae, M. catarrhalis*). Whilst other restriction enzymes have been shown to provide greater levels of resolution [26,27], no single restriction enzyme is able to resolve all bacterial species. Therefore, it must be recognised that in many instances, T-RF bands of the same length will be generated from different bacterial species. This may result in an underestimation of species richness, lower confidence in ascribing species to T-RF bands on the basis of T-RF band length alone, and an overestimation of the proportion of the total bacterial community represented by these bacterial species. Steps can be taken to offset the failure of a particular restriction enzymes to resolve all species present, including generating multiple profiles with different restriction enzymes, and such approaches may need to be applied were the techniques described here to be applied to a wider study of chronic respiratory infections.

SNA agar is routinely used to isolate yeasts from respiratory samples. Although the T-RFLP profiling used here was designed to resolve bacterial species alone, SNA cultures were included. The fact that they provided no signal in all but one instance indicated that this medium was highly selective for fungal species. Although no attempt was made to do so in this study, other studies have shown that fungal communities can be studied by Direct Molecular Analysis [28]. This would clearly be important to assess more generally in airway specimens.

This study also considered whether the bacteria detected were metabolically active. Unlike culture-based methods, the detection of bacteria in clinical samples by DNA-based methodologies does not indicate whether the bacteria in question are viable. Although activity does not necessarily imply pathogenicity, the presence of actively metabolising bacteria in sputum samples does suggest further investigation is warranted. The use of rRNA-based analyses to characterise active microbial communities is based on the assumption that active or growing cells have increased levels of rRNA relative to dormant, or intact dead cells. Similar comparisons have been made of bacteria in environments as diverse as soil [29], dairy fermentations [30] and CF sputum [18]. For these respiratory samples, approximately a quarter of the T-RF bands were detected in the direct analysis of sputum were found only in lanes generated from DNA, with 42% were found in both DNA and RNA and the remainder present in RNA alone. It is quite possible that those found only as a DNA signal were from inactive or dead cells. It is also possible that, given the variation in ribosomal operon numbers between bacterial species (from one to 15) [31-33], that this influenced the relative amounts of signal generated for any given band position. It should be noted that because rRNA transcripts are many times more common in bacterial cells than are rRNA genes, RNA-based analysis may provide a greater sensitivity in the detection of uncommon species within samples. This may well explain the significantly greater number of T-RF bands in RNA-derived profiles. Molecular based methods are not themselves without bias, with such bias known in the amplification process which will impact on both PCR and RT-PCR steps [34,35]. Despite this, whilst these data may be influenced by these technical issues, the findings suggest that three quarters of the bacteria present in these respiratory samples were metabolically active.

## Conclusion

There are still limitations in terms of molecular microbiology. For example, this study did not focus on species identification, rather on the degree to which culture-independent methodologies may preclude the identification of organisms that could have a causative association with a particular pathology or disease when applied to chronic respiratory conditions such as COPD or CF. Whilst some of the bacteria present in sputum samples may result from contamination during expectoration, it is also possible that they represent populations colonising the lower respiratory tract. Inclusive, culture-independent approaches, such those described here, provide a means by which further study could determine the degree to which these situations is the case.

Whilst culture-dependent diagnostics will continue to play an important role in the detection of known respiratory pathogens, the deployment of culture-independent profiling techniques will help to identify respiratory pathogens whose clinical significance is not yet recognised in these conditions. Clearly however, much more is needed to improve methodologies and more fundamentally understand the potential significance of the species detected in terms of airways disease. In particular, studies to determine whether the bacterial community profiles are stable over time, and how they change and respond to antimicrobial interventions, are now needed.

## Abbreviations used

(CF): Cystic Fibrosis; (COPD): Chronic Obstructive Pulmonary Disease; (T-RFLP): Terminal Restriction Fragment Length Polymorphism; (RT-T-RFLP): Reverse Transcriptase Terminal Restriction Fragment Length Polymorphism; (DMA): Direct Molecular Analysis; (CMA): Culture-derived Molecular Analysis; (RDM): Routine Diagnostic Microbiology.

## Competing interests

The authors declare that they have no competing interests.

## Authors' contributions

GBR performed all molecular microbiological analyses and wrote the initial draft of the paper. AT directed all conventional microbiology. TTD directed patient liason, sample collection and participated in study design. GBR, KDB and MPC conceived the question. KDB directed writing and analysis. KDB and MPC obtained funding for the project. GJC and GJPD participated in funding, data collection, data analysis and interpretation, and editing. All authors have read and approved the final manuscript.

## Pre-publication history

The pre-publication history for this paper can be accessed here:



## References

[B1] Wilson R (2001). Bacteria, antibiotics and COPD. Eur Respir J.

[B2] Miravitlles M (2002). Exacerbations of chronic obstructive pulmonary disease: when are bacteria important?. Eur Respir J.

[B3] Patel IS, Seemungal TA, Wilks M, Lloyd-Owen SJ, Donaldson GC, Wedzicha JA (2002). Relationship between bacterial colonisation and the frequency, character, and severity of COPD exacerbations. Thorax.

[B4] Hurst JR, Wilkinson TM, Perera WR, Donaldson GC, Wedzicha JA (2005). Relationships among bacteria, upper airway, lower airway, and systemic inflammation in COPD. Chest.

[B5] Donaldson GC, Seemungal TA, Patel IS, Bhowmik A, Wilkinson TM, Hurst JR, MacCallum PK, Wedzicha JA (2005). Airway and systemic inflammation and decline in lung function in patients with COPD. Chest.

[B6] Frederiksen B, Hoiby N, Koch C (1998). Age at onset of chronic pulmonary infection with P. aeruginosa infection is a predictor for survival in CF. Pediatric Pulmonology.

[B7] Frederiksen B, Koch C, Hoiby N (1997). Antibiotic treatment of initial colonization with Pseudomonas aeruginosa postpones chronic infection and prevents deterioration of pulmonary function in cystic fibrosis. Pediatric Pulmonology.

[B8] National Collaborating Centre for Chronic Conditions. Chronic obstructive pulmonary disease (2004). National clinical guideline on management of chronic obstructive pulmonary disease in adults in primary and secondary care. Thorax.

[B9] Smith AL, Fiel SB, Mayer-Hamblett N, Ramsey B, Burns JL (2003). Susceptibility testing of Pseudomonas aeruginosa isolates and clinical response to parenteral antibiotic administration – Lack of association in cystic fibrosis. Chest.

[B10] Foweraker JE, Laughton CR, Brown DF, Bilton D (2005). Phenotypic variability of Pseudomonas aeruginosa in sputa from patients with acute infective exacerbation of cystic fibrosis and its impact on the validity of antimicrobial susceptibility testing. J Antimicrob Chemother.

[B11] Amann RI, Ludwig W, Schleifer KH (1995). Phylogenetic identification and in situ detection of individual microbial cells without cultivation. Microbiol Rev.

[B12] Atlas RM, Bartha R (1992). Microbial ecology, fundamentals and applications.

[B13] Weng L, Rubin EM, Bristow J (2006). Application of sequence-based methods in human microbial ecology. Genome Res.

[B14] Liu WT, Marsh TL, Cheng H, Forney LJ (1997). Characterization of microbial diversity by determining terminal restriction fragment length polymorphisms of genes encoding 16S rRNA. Appl Environ Microbiol.

[B15] Rogers GB, Hart CA, Mason JR, Hughes M, Walshaw MJ, Bruce KD (2003). Bacterial diversity in cases of lung infection in cystic fibrosis patients: 16S ribosomal DNA (rDNA) length heterogeneity PCR and 16S rDNA terminal restriction fragment length polymorphism profiling. J Clin Microbiol.

[B16] Rogers GB, Carroll MP, Serisier DJ, Hockey PM, Jones G, Bruce KD (2004). Characterization of bacterial community diversity in cystic fibrosis lung infections by use of 16S ribosomal DNA terminal restriction fragment length polymorphism profiling. J Clin Microbiol.

[B17] Rogers GB, Carroll MP, Serisier DJ, Hockey PM, Jones G, Bruce KD (2005). Bacterial activity in cystic fibrosis lung infections. Respir Res.

[B18] Health Protection Agency (2008). Investigation of bronchoalveolar lavage, sputum and associated specimens, BSOP 57.

[B19] Rogers GB, Carroll MP, Serisier DJ, Hockey PM, Jones G, Kehagia V, Connett GJ, Bruce KD (2006). Use of 16S rRNA gene profiling by terminal restriction fragment length polymorphism analysis to compare bacterial communities in sputum and mouthwash samples from patients with cystic fibrosis. J Clin Microbiol.

[B20] Duan K, Dammel C, Stein J, Rabin H, Surette MG (2003). Modulation of Pseudomonas aeruginosa gene expression by host microflora through interspecies communication. Mol Microbiol.

[B21] Keller L, Surette MG (2006). Communication in bacteria: an ecological and evolutionary perspective. Nat Rev Microbiol.

[B22] Worlitzsch D, Tarran R, Ulrich M, Schwab U, Cekici A, Meyer KC, Birrer P, Bellon G, Berger J, Weiss T, Botzenhart K, Yankaskas JR, Randell S, Döring G (2002). Effects of reduced mucus oxygen concentration in airway Pseudomonas infections of cystic fibrosis patients. J Clin Invest.

[B23] Yoon SS, Hennigan RF, Hilliard GM, Ochsner UA, Parvatiyar K, Kamani MC, Allen HL, DeKievit TR, Gardner PR, Schwab U, Rowe JJ, Iglewski BH, McDermott TR, Mason RP, Wozniak DJ, Hancock RE, Parsek MR, Noah TL, Boucher RC, Hassett DJ (2002). Pseudomonas aeruginosa anaerobic respiration in biofilms: Relationships to cystic fibrosis pathogenesis. Dev Cell.

[B24] Kolak M, Karpati F, Monstein HJ, Jonasson J (2003). Molecular typing of the bacterial flora in sputum of cystic fibrosis patients. Int J Med Microbiol.

[B25] Alarcon B, Vicedo B, Aznar R (2006). PCR-based procedures for detection and quantification of Staphylococcus aureus and their application in food. J Appl Microbiol.

[B26] Moyer CL, Tiedje JM, Dobbs FC, Karl DM (1996). A computer-simulated restriction fragment length polymorphism analysis of bacterial small-subunit rRNA genes: efficacy of selected tetrameric restriction enzymes for studies of microbial diversity in nature. Appl Environ Microbiol.

[B27] Engebretson JJ, Moyer CL (2003). Fidelity of select restriction endonucleases in determining microbial diversity by terminal-restriction fragment length polymorphism. Appl Environ Microbiol.

[B28] Singh BK, Nazaries L, Munro S, Anderson IC, Campbell CD (2006). Use of multiplex terminal restriction fragment length polymorphism for rapid and simultaneous analysis of different components of the soil microbial community. Appl Environ Microbiol.

[B29] Pesaro M, Nicollier G, Zeyer J, Widmer F (2004). Impact of soil drying-rewetting stress microbial communities and activities and on degradation of two crop protection products. Appl Environ Microbiol.

[B30] Sanchez JI, Rossetti L, Martinez B, Rodríguez A, Giraffa G (2006). Application of reverse transcriptase PCR-based T-RFLP to perform semi-quantitative analysis of metabolically active bacteria in dairy fermentations. J Microbiol Methods.

[B31] Bercovier H, Kafri O, Sela S (1986). Mycobacteria possess a surprisingly small number of ribosomal RNA genes in relation to the size of their genome. Biochem Biophys Res Commun.

[B32] Andersson SG, Zomorodipour A, Winkler HH, Kurland CG (1995). Unusual organization of the rRNA genes in Rickettsia prowazekii. J Bacteriol.

[B33] Rainey FA, WardRainey NL, Janssen PH, Hippe H, Stackebrandt E (1996). Clostridium paradoxum DSM 7308(T) contains multiple 16S rRNA genes with heterogeneous intervening sequences. Microbiology-Uk.

[B34] Suzuki MT, Giovannoni SJ (1996). Bias caused by template annealing in the amplification of mixtures of 16S rRNA genes by PCR. Appl Environ Microbiol.

[B35] Polz MF, Cavanaugh CM (1998). Bias in template-to-product ratios in multitemplate PCR. Appl Environ Microbiol.

